# Optimized Neural Network Prediction Model of Shape Memory Alloy and Its Application for Structural Vibration Control

**DOI:** 10.3390/ma14216593

**Published:** 2021-11-02

**Authors:** Meng Zhan, Junsheng Liu, Deli Wang, Xiuyun Chen, Lizhen Zhang, Sheliang Wang

**Affiliations:** 1 College of Construction Engineering, HuangHuai University, No. 76 Kaiyuan RD., Zhumadian 463000, China; Cxy0396@126.com (X.C.); zhenzhen_5255@sina.com (L.Z.); 2 Shaanxi Institute of Building Science, No. 272 Huancheng RD., Xi’an 710082, China; jsliu1973@163.com; 3 Taizhou Urban and Rural Planning and Design Institute, No. 465 Shifu RD., Taizhou 318000, China; wangdeli2012@sina.com; 4 College of Civil Engineering, Xi’an University of Architecture and Technology, No. 13 Yanta RD., Xi’an 710055, China; sheliangw@163.com

**Keywords:** SMA, prediction model, BP neural network, genetic algorithm, seismic response

## Abstract

The traditional mathematical model of shape memory alloy (SMA) is complicated and difficult to program in numerical analysis. The artificial neural network is a nonlinear modeling method which does not depend on the mathematical model and avoids the inevitable error in the traditional modeling method. In this paper, an optimized neural network prediction model of shape memory alloy and its application for structural vibration control are discussed. The superelastic properties of austenitic SMA wires were tested by experiments. The material property test data were taken as the training samples of the BP neural network, and a prediction model optimized by the genetic algorithm was established. By using the improved genetic algorithm, the position and quantity of the SMA wires were optimized in a three-storey spatial structure, and the dynamic response analysis of the optimal arrangement was carried out. The results show that, compared with the unoptimized neural network prediction model of SMA, the optimized prediction model is in better agreement with the test curve and has higher stability, it can well reflect the effect of loading rate on the superelastic properties of SMA, and is a high precision rate-dependent dynamic prediction model. Moreover, the BP network constitutive model is simple to use and convenient for dynamic simulation analysis of an SMA passive control structure. The controlled structure with optimized SMA wires can inhibit the structural seismic responses more effectively. However, it is not the case that the more SMA wires, the better the shock absorption effect. When SMA wires exceed a certain number, the vibration reduction effect gradually decreases. Therefore, the seismic effect can be reduced economically and effectively only when the number and location of SMA wires are properly configured. When four SMA wires are arranged, the acceptable shock absorption effect is obtained, and the sum of the structural storey drift can be reduced by 44.51%.

## 1. Introduction

Shape memory alloy (SMA) is a novel functional material, which not only has two unique properties of shape memory and super elasticity, but also has the advantages of high damping, fatigue, and corrosion resistance [1,2,3]. Therefore, SMA has been widely used in the field of civil engineering for reinforcement and vibration control [4,5,6]. Dehghani and Aslani developed hooked-end pseudoelastic shape memory alloy fibres (PSMAF) which can provide re-centring and crack-closing behaviour in cementitious composites [7]. Rezapour et al. investigated the effects of SMA post-tensioning reinforcements on originally unreinforced masonry walls using Abaqus soft [8]. Siddiquee et al. evaluated the seismic performance of concrete frame buildings reinforced with superelastic SMA rebar in terms of the collapse margin ratio, and the results are compared with that of a reinforced concrete structure with regular steel rebar only [9]. Schranz et al. studied the effectiveness of two different prestressed strengthening methods for flexural behavior of concrete members using ribbed Fe-SMA bars [10]. Peng et al. proposed a shape memory alloy cable-double friction pendulum bearing which combines a double friction pendulum bearing with superelastic SMA cables [11]. Based on the pseudoelasticity of SMA and the electrodeformation of piezoelectric transition ceramic, Zhan et al. designed a novel SMA/PZT composite control device and investigated its energy dissipation performance and neural network constitutive model [12]. In addition, some researchers discussed the control of shape memory alloy actuator using PID controller and pulse width modulation [13,14,15,16].

The constitutive model of SMA material is the basis of theoretical analysis and experimental study for properties of SMA [17,18]. A series of constitutive models of SMA have been established based on material experiments. Falk established a theoretical constitutive model of a single crystal based on Landau’s theory, but it only applies to single crystal and only considers the shearing motion of crystal [19]. Abeyartane and Knowle proposed a one-dimensional constitutive model of SMA material by using the thermodynamic bar theory and combining the Helmholzt free energy principle and the expression of thermodynamic relation, but this model did not consider the redirection of stress-induced martensite variation under non-proportional load [20]. Boyd and Lagoudas presented a meso-mechanical constitutive model based on meso-mechanics and thermodynamics, but this model is too complex to be applied in engineering [21]. Based on the constitutive relationship between thermodynamics and kinetics, Brinson proposed a phenomenological constitutive model with the characteristics of plasticity theory, which is the most widely used constitutive model of SMA [22]. Liu et al. explored a macroscopic phenomenological constitutive model involving strain amplitude and loading rate by introducing an internal variable evolution equation, considering the difference of characteristic parameters during the positive/reverse transformation of martensite and the hardening properties of martensite under large strain amplitudes [23]. Based on the Landau theory of phase transitions, Du et al. proposed a differential model to describe the hysteresis of magnetic shape memory alloys caused by magnetic field induced martensite reorientation [24]. However, for shape memory alloy, there are many factors affecting its constitutive model, so it is impossible to establish an accurate mathematical model to express the influence of all various factors on it, and it can only be simplified and approximated artificially. The artificial neural network does not need to achieve the precise constitutive relation of the material, but only needs to consider the influencing factors and expected objectives, and it avoids the inevitable error in the traditional modeling method and provides a new way to establish the accurate SMA constitutive model. Lee and Lee used a multi-layer perceptron neural network to forecast the resilience of SMA [25]. Ren et al. used a radial basis function network to forecast the hysteresis behavior of SMA at the same strain amplitude with the number of cycles, loading information, and strain value as the input of neurons [26]. However, the change of initial weight/threshold of neurons has a great influence on the prediction results of the artificial neural network, so the initial weight/threshold should be optimized in the neural network to improve the accuracy and stability.

The damping effect of the SMA damper on an engineering structure is mainly determined by its own performance, installation position, and layout number [27,28]. Even if its damping force is large and the shock absorption effect is good, the ideal damping effect may not be achieved if the installation position and number are inappropriate [29,30,31]. Therefore, in order to achieve the efficiency and economy of the SMA control system, it is necessary to optimize the installation position and quantity of SMA wires in the engineering application. By idealizing the hysteretic curve of SMA damper and the transient tangent stiffness matrix of structure, Mulay and Shmerling presented a simplified nonlinear equation model to design SMA dampers in three-dimensional asymmetric plane structures [32]. Pang et al. proposed a novel seismic risk-based optimization design method of SMA restrained sliding bearings in highway bridges suffered from near-fault ground motions via the particle swarm optimization [33]. Zhan et al. discussed the optimal arrangement of SMA piezoelectric composite dampers in a space truss structure using the adaptive immune memory cloning algorithm with the performance indicator based on the modal controllable degree as antigen-antibody affinity function [34].

This paper explored a novel SMA prediction model considering loading rate and loading history via a back-propagation (BP) neural network optimized by the genetic algorithm (GA). SMA wires were selected to control the dynamics response of a three-storey spatial structure, and the application of artificial neural network to SMA constitutive model and the damping effect of SMA wires on the seismic response of spatial structure are discussed. Firstly, the sum of lateral inter-story displacement is taken as the control objective, the optimization of the number and position of SMA wires is carried out by applying the improved genetic algorithm. Then, the Newmark-β method is employed to accomplish the seismic response control analysis of the spatial structure with an optimal allocation of SMA wires via MATLAB soft.

## 2. SMA Wire Mechanical Performance Test

### 2.1. Test Loading Scheme

The chemical composition of the austenitic SMA wire used in the test is Ti-51%atNi [35,36] with diameters 0.5, 0.8, 1.0, and 1.2 mm. Phase transition temperature: Martensite finish temperature *M*_f_ is −42 °C; Martensite start temperature *M*_s_ is −38 °C; Austenite start temperature *A*_s_ is −6 °C; Austenite finish temperature *A*_f_ is −2 °C. Taiwan Hongda HT-2402 computer servo-controlled material testing machine is used in Material Science Laboratory of Xi’an University of Technology. The axial force is measured by the force sensor that comes with the testing machine, and the axial deformation is measured by the displacement extensometer. The gauge length is 33.5 mm, and all data are automatically collected by computer.

This test mainly considers the influence of the number of loading cycles, strain amplitude, loading rate, and material diameter on the stress–strain curve, energy dissipation capacity, equivalent damping ratio, and equivalent secant modulus of austenitic SMA wire. In this experiment, the loading rate was 10 mm/min, 30 mm/min, 60 m/min, 90 mm/min, and the strain amplitude was 3%, 6%, and 8%. To ensure the accuracy of the test, a pre-tension of 10~30 MPa is applied to the SMA wire before each working condition starts to work so that the SMA wire can be straightened and tightened. To eliminate the influence of the test piece length on SMA, the length of the test piece used is 300 mm with an effective length of 100 mm. In each cycle, only when the SMA wire strain reaches the amplitude strain, loading stops, and when the axial force of the SMA wire was less than 5N, unloading stops. Loading cycle was 30 cycles in each working condition.

### 2.2. Test Results and Analysis

**Figure 1:**
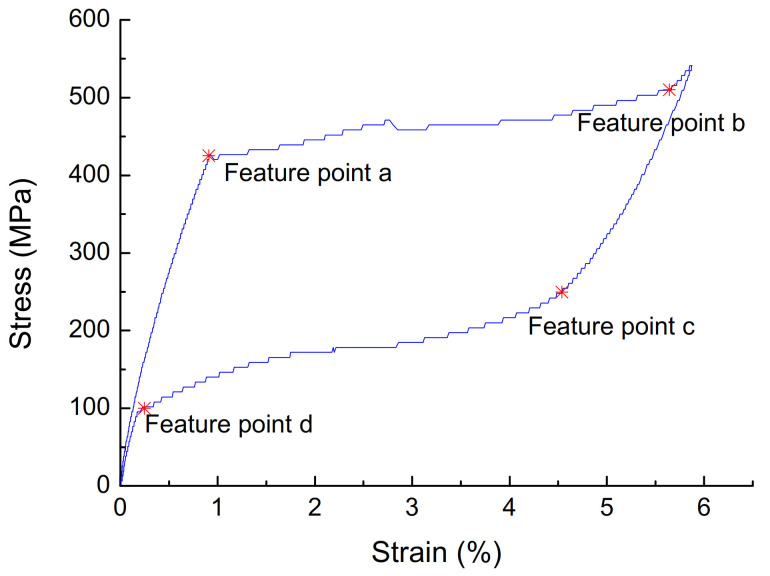
Characteristic points of austenite SMA constitutive curve.

The stress of four points (*σ_a_*, *σ_b_*, *σ_c_*, *σ_d_*) is used to replace the critical stress of SMA transformation, as shown in Figure 1. The starting point of the stress–strain curve platform of the loading section is taken as point a. The point where the slope of the loading section curve is significantly increased after the loading platform is taken as point b. The point where the stress–strain drops start to deviate from the linear relation is taken as point c. The point where the stress and strain begin to decrease at the end is taken as point d. Substituting the above-mentioned points for the phase transition critical point of austenite SMA, although there is a certain error between the characteristic point stress and the true phase transition stress of SMA, the phase transition process of SMA can still be reflected.

The mechanical performance indicators are defined as follows: Δ*W* represents the energy consumption value of the SMA wire in a single cycle, that is, the graphic area enclosed by the stress–strain curve; *K*_s_ represents the equivalent secant stiffness of a single cycle; *δ* represents the equivalent damping ratio of a single cycle.

(1) $$K_{s}=\frac{\sigma_{\max}-\sigma_{\min}}{\varepsilon_{\max}-\varepsilon_{\min}}$$

(2) $$\delta =\frac{\Delta W}{2\pi K_{s}\varepsilon^{2}}$$

where *σ*_max_ (*ε*_max_) and *σ*_min_ (*ε*_min_) respectively represent the maximum stress (strain) and minimum stress (strain) of each cycle, and *ε* is the amplitude strain.

According to the experimental results, the effects of cyclic loading, strain amplitude, loading rate and material diameter on the mechanical properties of austenitic SMA wire are analyzed.

**Figure 2:**
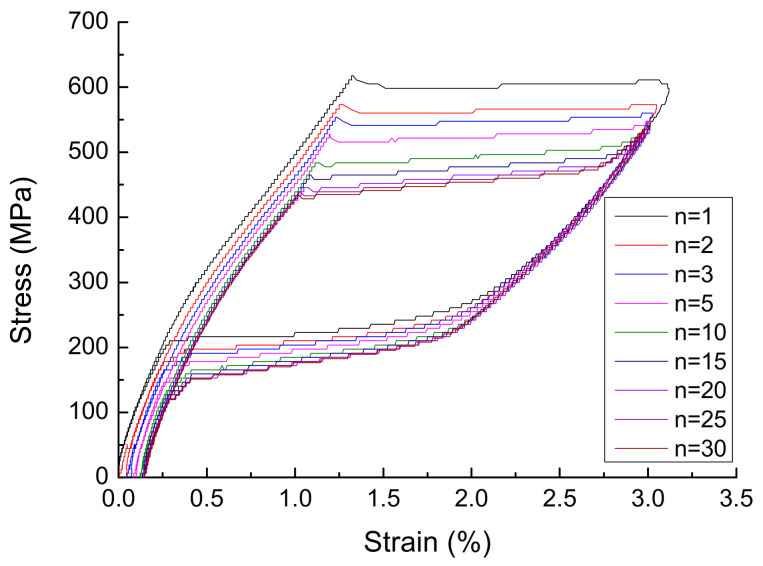
Stress-strain curve of austenite SMA wire with different cycles.

**Figure 3:**
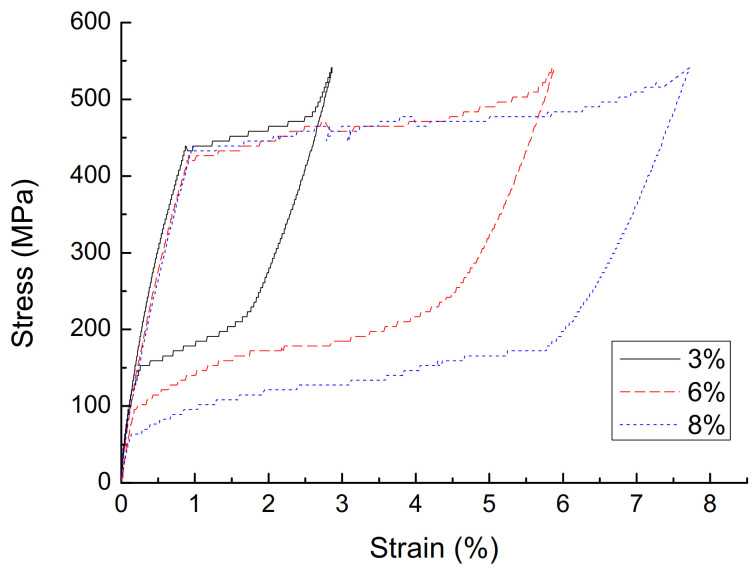
Stress-strain curve of austenite SMA wire with different strain amplitudes.

**Figure 4:**
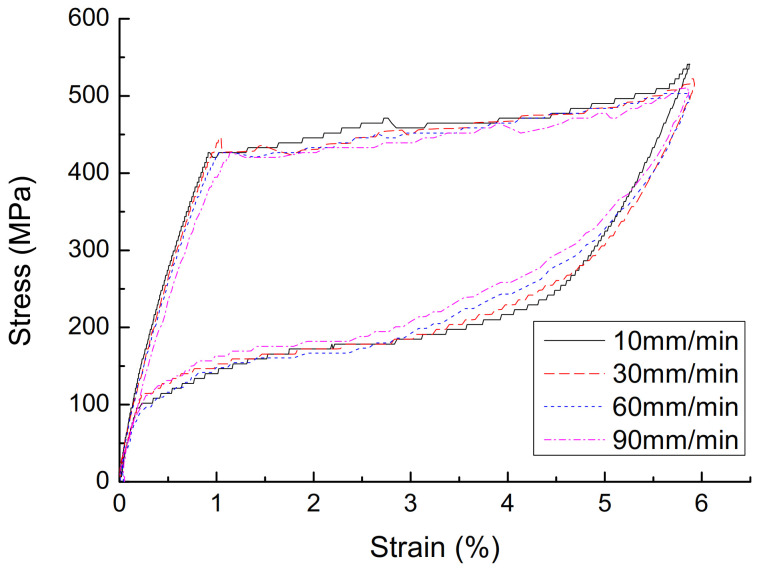
Stress-strain curve of austenite SMA wire with different loading rates.

**Figure 5:**
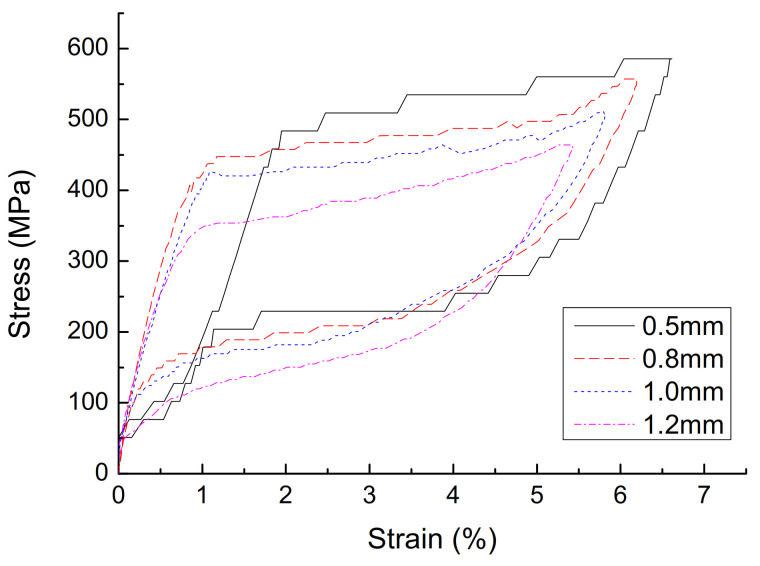
Stress-strain curve of austenite SMA wire with different diameters.

(1) The effect of cyclic loading on the mechanical properties of superelasticity. The stress–strain curve with a diameter of 1.0 mm, a loading rate of 10 mm/min, and a strain amplitude of 3% is shown in Figure 2, and the parameters are shown in Table 1. It can be seen that, with the increase of the number of cycles, the cumulative residual deformation of the austenitic SMA wire gradually increases, but the residual deformation of the single cycle gradually becomes smaller, and stabilizes after 15 cycles, and the residual strain is zero. With the increase in the number of cycles of loading, the performance of austenitic SMA wire gradually stabilizes, the stress–strain curve gradually becomes smooth, the energy dissipation capacity and equivalent damping ratio of the SMA wire gradually decrease, and the equivalent secant stiffness slightly decreased, but stabilized after 15 loading/unloading cycles. The number of cycles has a great influence on the mechanical properties of austenitic SMA wire. In actual engineering applications, in order to obtain stable superelastic properties, the SMA wire must be cyclically loaded in advance, and it usually takes about 20 cycles.
(2) The effect of strain amplitude on the mechanical properties of superelasticity. A stress–strain curve with a diameter of 1.0 mm, a loading rate of 10 mm/min, and a strain amplitude of 3% is shown in Figure 3, and the parameters are shown in Table 2. As the strain amplitude of SMA wire increases, the cumulative residual deformation gradually increases. When the strain amplitude is small, the austenitic SMA wire is basically in the elastic stage, and the elastic modulus is approximately 450 MPa after stabilization. When the strain amplitude exceeds 1%, the SMA wire will undergo martensitic transformation and austenite transformation, showing super-elastic performance, and the greater the strain amplitude, the better its super-elastic performance, and the greater the energy dissipation capacity. The strain amplitude is the most significant factor affecting the energy dissipation capacity of SMA wires. When the strain amplitude increases from 3% to 8%, the single-turn energy consumption of the SMA wire increases from 4.46 MJ·m^−3^ to 20.76 MJ·m^−3^, which increases the energy consumption by 3.65 times. As the strain amplitude increases, the damping ratio gradually increases, the equivalent secant stiffness gradually decreases, and the energy consumption capacity continues to increase.
(3) The effect of loading rate on the mechanical properties of superelasticity. The stress–strain curve of the 30th cycle with a diameter of 1.0 mm, and a strain amplitude of 6% is shown in Figure 4, and the parameters are shown in Table 3. As the loading rate increases, the single-cycle energy consumption of the austenitic SMA wire gradually decreases, and the shape of the stress–strain curve changes significantly. In the phase of unloading, the initial stress of the change increases significantly. The stress–strain shape gradually transitions from a rectangle and a diamond to a trapezoid and a narrower triangle. The area enclosed by the hysteresis curve gradually decreases. The equivalent damping ratio and equivalent stiffness generally show a decreasing trend and energy consumption gradually decreases. This is mainly because the heat generated during the loading process of the SMA wire causes the temperature rise of the SMA specimen, which reduces its own energy consumption.
(4) The effect of diameters on the mechanical properties of superelasticity. The stress–strain curve of the 30th cycle with a loading ratio of 90 mm, and a strain amplitude of 6% is shown in Figure 5, and the parameters are shown in Table 4. As the diameter of the material increases, the stress–strain curve of the SMA wire tends to be smooth, but the number of cycles required to reach stability increases, and the cumulative residual deformation presents a gradually increasing trend. The stress of each characteristic point of SMA wire decreases with the increase of the material diameter. As the diameter of the material increases, the energy dissipation capacity and equivalent damping ratio show a significant decrease. This is mainly due to the increase in the diameter of the material, and the heat generated during the loading process cannot be dissipated in time, causing the specimen temperature to increase, which reduces the energy consumption of SMA wire. The equivalent stiffness is less affected by the diameter, and the change is not obvious. Therefore, in engineering applications, SMA wires with appropriate diameters should be selected for the passive control of seismic response.

## 3. BP Neural Network Model Optimized by GA

**Figure 6:**
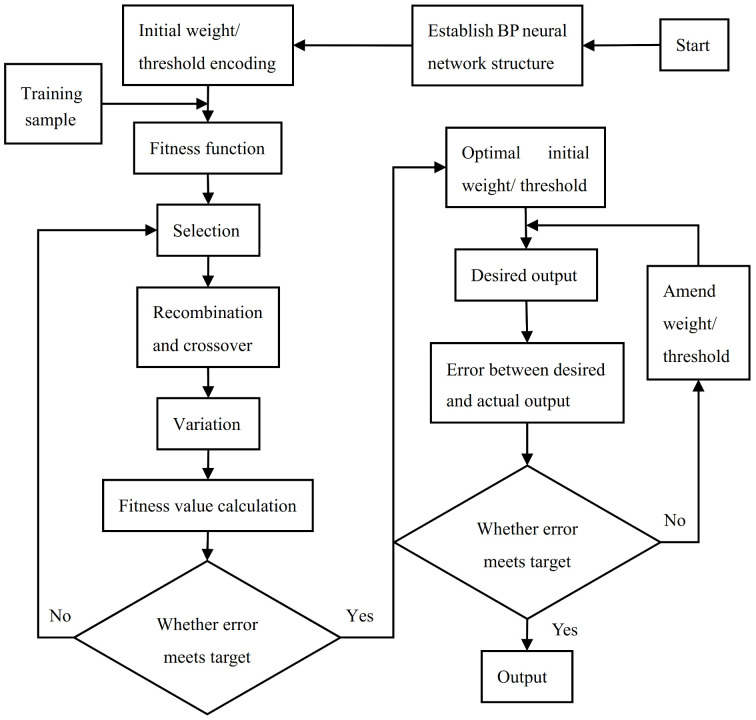
Flow chart of BP network optimized by genetic algorithm.

The BP neural network is a multi-layer feedforward network composed of input layer, hidden layer, and output layer [37,38]. The initial weights/thresholds are randomly selected by system that are different between different each training. Therefore, the final weights/thresholds and models of the neural network obtained after training are different, especially when the training data are small. It is possible that the two neural network models are completely different, so the generalization ability of neural network is poor. When the training data are sufficient and extensive, although the difference between the neural network models after training is small, the training convergence speed will be too low. Using the genetic algorithm to search for the best initial weight/threshold value in the entire range of weight/threshold value, the error of BP neural network after training under the best initial weight/threshold value can be minimized. In this way, the difference of the BP neural network after training due to different initial weights/thresholds can be avoided, and the problem of network oscillation and non-convergence caused by improper initial weights/threshold values can also be prevented [39,40]. The process of optimizing the initial weight/threshold of the BP neural network by GA is shown in Figure 6.

### 3.1. Structure of BP Neural Network

(1) Number of neurons in the input layer: When the diameter of the SMA wire is constant, the SMA constitutive relationship after stable performance is mainly affected by the loading rate and loading history. Therefore, the following variables can be determined as the input neurons of the BP neural network:
  (3) $$x_{1}=v,\;x_{2}=\sigma_{t-2},\;x_{3}=\varepsilon_{t-2},\;x_{4}=\sigma_{t-1},\;x_{5}=\varepsilon_{t-1},\;x_{6}=\varepsilon_{t}$$
  where *v* is the loading rate of the SMA wire, *σ_i_* and *ε_i_* represent the stress and strain at time *I*, respectively.
(2) Number of neurons in the output layer: The variable required by the SMA constitutive model is the stress *σ _t_* at time t, so *y = σ_t_* is determined as the output neuron of the BP neural network.
(3) Number of neurons in the hidden layer: The number of neurons in the hidden layer is a complex problem to be solved in the BP neural network. Currently, estimation methods [12] are usually used to determine the number of neurons in the hidden layer, and that is taken as 20.
(4) Neuron activation function: The activation function of the hidden layer neuron of the BP neural network is selected as logsig, and the activation function of the output layer neuron is selected as purelin.

### 3.2. Training Sample Collection and Processing

First, the training data is collected based on the test data of SMA wires. Since the SMA wire with a diameter of 1.0 mm is selected for structural vibration control, the neural network constitutive model for SMA with a diameter of 1.0 mm should be established. There are 48 working conditions in the SMA material property test, among which there are 12 working conditions with a diameter of 1.0 mm, and 4 of them are selected as the inspection data, respectively: ① The diameter is 1.0 mm, the loading rate is 10 mm/min, and the strain amplitude is 6%; ② The diameter is 1.0 mm, the loading rate is 30 mm/min, and the strain amplitude is 6%; ③ The diameter is 1.0 mm, the loading rate is 60 mm/min, and the strain amplitude is 6%; ④ The diameter is 1.0 mm, the loading rate is 90 mm/min, and the strain amplitude is 6%. The remaining 8 working conditions are for training data.

Then, the training data is normalized to obtain the samples required for training the BP neural network. Since the hidden layer neurons use the sigmoid activation function that is a saturation zone near the function value 0 and 1, and the function value changes very slowly, the normalization process can prevent the input data from entering the saturated region due to excessive absolute value.

### 3.3. Optimization Parameters of GA

The initial weight/threshold value of the unoptimized BP neural network is randomly assigned by the system, and that of the optimized BP neural network is determined by GA. According to the BP neural network structure, the weights to be determined in the BP neural network are 6 × 20 + 20 × 1 = 140, and there are 20 + 1 = 21 thresholds to be determined. The variables of GA are weights and thresholds, so the total number of variables is 161. Since the weight/threshold value can be any real number, in order to improve the accuracy and efficiency of the genetic algorithm, real-valued coding is used, and the chromosome length of the genetic algorithm is 140 + 21 = 161. The objective function is the sum of squared errors between the expected output and the actual output obtained from the input of the training sample. Other parameters of GA: the initial population number is 40; the random traversal sampling selection function is used, and the generation gap is 0.9; the intermediate recombination crossover operator is selected; the real-valued mutation operator is used, and the mutation probability is 0.01; the maximum genetic algebra is 50.

### 3.4. Simulation Results and Analysis

**Figure 7:**
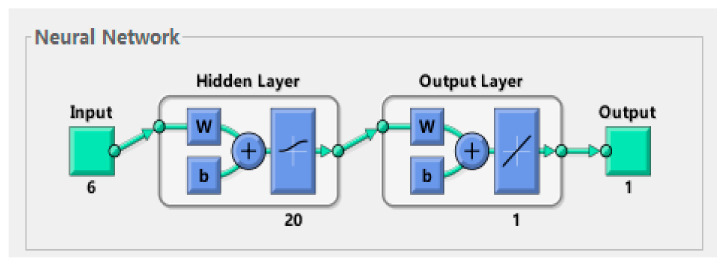
BP neural network topology.

**Figure 8:**
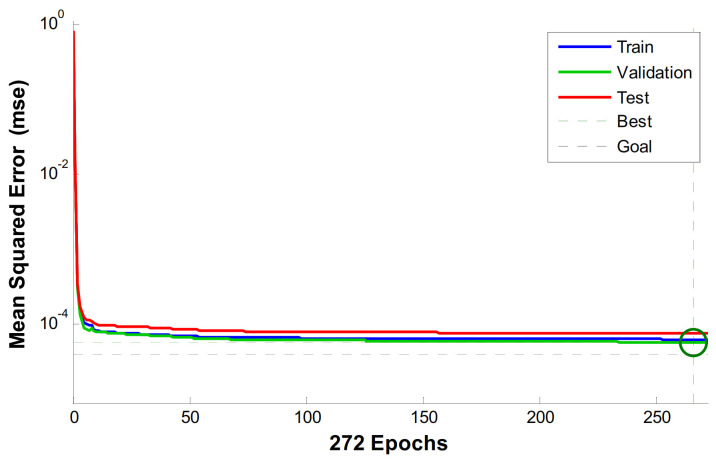
Validation Performance Chart.

The first step is to create a BP neural network model of austenite SMA, and a BP neural network model optimized by GA. MATLAB2013b neural network toolbox and gatbx toolbox are used to write simulation program code. The data of the last cycle of the test condition are used as the training sample of BP neural network. The training function is selected as trainlm, the maximum number of training times is 1000, the target error is 10^−5^, and the learning rate is 0.1. The topological structure of BP neural network can be obtained by running the program as shown in Figure 7, and the validation performance chart is shown in Figure 8.

The second step is to compare the accuracy of unoptimized and optimized BP neural network models. Four groups of normalized inspection data are input into the unoptimized BP neural network and the optimized BP neural network in sequence, and the expected output is obtained respectively. The accuracy of the two constitutive models is obtained by comparing expected output with the actual output and error analysis.

**Figure 9:**
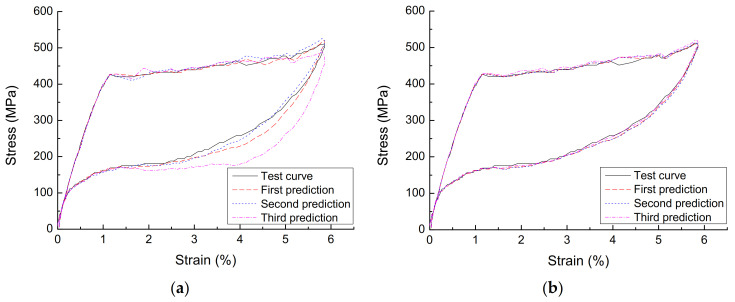
Comparison of BP network prediction curve and test curve (**a**) unoptimized BP network and (**b**) optimized BP network.

Figure 9 is a comparison diagram of the BP neural network prediction curve and the experimental curve at the diameter of 1.0 mm, loading rate of 90 mm/min, and strain amplitude of 6%. It can be seen that, after each training, the BP neural network prediction curve without GA optimization has a large fluctuation range due to the randomness of the initial weight/threshold, but that optimized by GA is in good agreement with the test curve, avoiding the difference of the model obtained from each run of the BP neural network, and it is a constitutive model of SMA with good stability and high accuracy.

**Figure 10:**
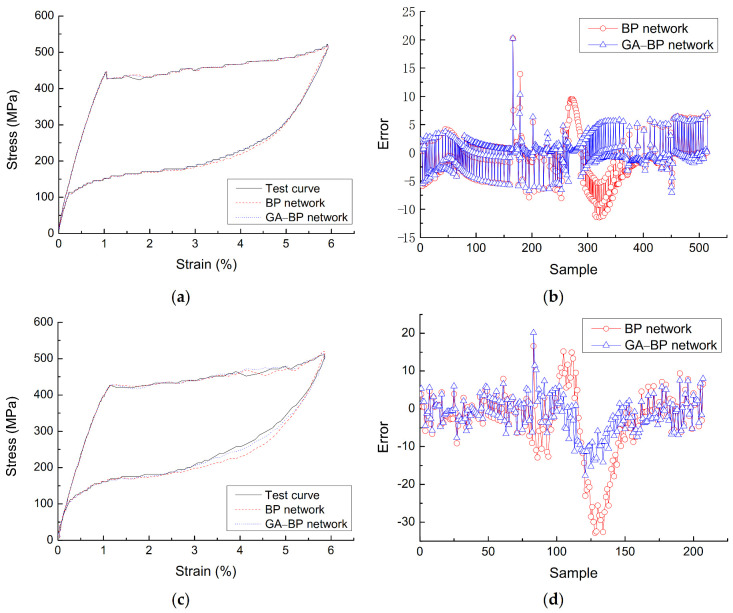
Comparison and corresponding error between test curve and prediction curves of un-optimized and optimized BP network by GA, (**a**) constitutive curves at 30 mm/min, (**b**) error at 30 mm/min, (**c**) constitutive curves at 90 mm/min, (**d**) error at 90 mm/min.

Since the network structure of the unoptimized BP neural network is different each time, the unoptimized BP neural network with better prediction results is selected for comparison with the optimized BP neural network. Figure 10 shows the comparison and error of the test curve with the unoptimized and optimized neural network prediction curve under different loading rates when the diameter is 1.0 mm and the loading amplitude is 6%. The average absolute percentage error *E*_P_ and *E*_GP_ of the prediction results of the two BP neural network models at the loading rate of 90 mm/min are:

(4) $$E_{P}=\frac{1}{n}\overset{n}{\underset{i=1}{\sum }}\frac{\left|Y_{i}-Y_{Pi}\right|}{Y_{i}}=2.72\%$$

(5) $$E_{\mathrm{GP}}=\frac{1}{n}\overset{n}{\underset{i=1}{\sum }}\frac{\left|Y_{i}-Y_{\mathrm{GP}i}\right|}{Y_{i}}=2.13\%$$

The linear coefficient of correlation γP and γGP of two prediction results are:

(6) $$\gamma_{P}=\frac{\sum_{i=1}^{n}\left(Y_{i}-\bar{Y}\right)\left(Y_{Pi}-{\bar{Y}}_{P}\right)}{\sqrt{\sum_{i=1}^{n}{\left(Y_{i}-\bar{Y}\right)}^{2}\cdot \sum_{i=1}^{n}{\left(Y_{Pi}-{\bar{Y}}_{P}\right)}^{2}}}=0.9983$$

(7) $$\gamma_{\mathrm{GP}}=\frac{\sum_{i=1}^{n}\left(Y_{i}-\bar{Y}\right)\left(Y_{\mathrm{GP}i}-{\bar{Y}}_{\mathrm{GP}}\right)}{\sqrt{\sum_{i=1}^{n}{\left(Y_{i}-\bar{Y}\right)}^{2}\cdot \sum_{i=1}^{n}{\left(Y_{\mathrm{GP}i}-{\bar{Y}}_{\mathrm{GP}}\right)}^{2}}}=0.9995$$

The root mean square error *σ*_P_ and *σ*_GP_ of two prediction results are:

(8) $$\sigma_{P}=\sqrt{\frac{\sum_{i=1}^{n}{\left(Y_{i}-Y_{Pi}\right)}^{2}}{n}}=9.76$$

(9) $$\sigma_{\mathrm{GP}}=\sqrt{\frac{\sum_{i=1}^{n}{\left(Y_{i}-Y_{\mathrm{GP}i}\right)}^{2}}{n}}=5.43$$

where $Y_{i}$ and $\bar{Y}$ are the sample and mean stress measured in the experiment, $Y_{Pi}$ and ${\bar{Y}}_{P}$ are the sample and mean stress predicted by the BP neural network model, and $Y_{\mathrm{GP}i}$ and ${\bar{Y}}_{\mathrm{GP}}$ are the sample and mean stress predicted by the BP neural network model optimized by GA.

The analysis shows that: (a) With the change of SMA wire loading rate, the optimized BP neural network model can track the force behavior of SMA well, and the average absolute percentage error is 2.13%, the linear coefficient of correlation is 0.9995 and the root mean square error is 5.43. This shows that the neural network constitutive model based on GA optimization can well describe the change of SMA superelastic performance with loading rate, better predict the superelastic restoring force of SMA under repeated loading, and is a good rate-dependent dynamic constitutive model. (b) Although the unoptimized BP constitutive curve with better prediction effect is compared with the BP constitutive curve optimized by GA, the accuracy of the optimized model is still higher than that of the unoptimized model. The error distribution of BP constitutive curve optimized by GA and the experimental constitutive curve is relatively concentrated. Only the error of individual sample points is far from 0, and the absolute maximum error is smaller than that of the unoptimized BP network. The error distribution between the unoptimized neural network curve and the experimental curve is relatively scattered. More importantly, the optimal initial weight/threshold value obtained by optimization replaces the random value assigned by the system, so that the BP neural network has a fixed optimal initial weight/threshold value, avoiding the difference of the BP neural network after each training.

## 4. Optimization Control of Spatial Structure with SMA Wires

### 4.1. Dynamic Equation of SMA Passive Control System

According to the basic theory of structural dynamics [41,42], the motion equation of a structure equipped with an austenitic SMA passive control system under seismic excitation can be expressed as:

(10) $$M\ddot{x}+C\dot{x}+Kx=-MI{\ddot{x}}_{g}+u$$

where ${\ddot{x}}_{g}$ is the acceleration of ground motion under seismic excitation; *M*, *K*, and *C* are the mass matrix, stiffness matrix and damping matrix of the structure, respectively; $\ddot{x},\;\dot{x},\;\mathrm{and}\;x$ are the acceleration, velocity and displacement column vector of the structure relative to the ground; I is the unit column vector; u is the passive control force column vector of the SMA wires acting on the structural nodes.

(11) $$u={\left\{u_{1}{}^{T},u_{2}{}^{T}\cdots u_{n}{}^{T}\right\}}^{T}$$

(12) $$\left\{\begin{array}{c} u_{i}=\sum_{j}\left\{F_{ij}\cdot \cos\theta_{ij}\right\}\;\;\;\;\;\varepsilon_{ij}>0\;\\ u_{i}=0\;\;\;\;\;\;\;\;\;\;\;\;\;\;\;\;\;\;\varepsilon_{ij}\leq 0\; \end{array}\right.$$

(13) $$F_{ij}=\sigma_{ij}\cdot A_{ij}$$

where $u_{i}$ is the control force column vector of SMA wire acting on the structure node *i*; $\theta_{ij}$ represents the direction cosine column vector of the *j*-th SMA wire connected to the structural node *i* and the coordinate axes X, Y, and Z; *F_ij_* represents the tension of the *j-*th SMA wire connected to the structural node *i*; *σ_ij_*, *ε_ij_*, and *A_ij_* refer to the tensile stress, tensile strain and cross-sectional area of the *j-*th SMA wire connected to the structural node *i.*

MATLAB is used to compile the Newmark-β method calculation program to analyze the dynamic time history of the space model structure with the SMA passive control system. The simulation time step interval is very small, and the earthquake action is also very small in the first two-time steps, that is, the force of the SMA wire is very small in the initial stage. Therefore, it is approximately considered that in the first two-time steps of the earthquake, the *σ_ij_* and *ε_ij_* of all SMA wires are 0, and the structure is in an uncontrolled state. Stress σ*_ij_* at any time later can be obtained via SMA BP network constitutive model optimized by GA. The vibration velocity at time *t*, the stress and strain at time *t −* 1 and *t −* 2, and the strain at time *t* are input into the optimized BP network constitutive model. Then, the stress of the SMA wire at time *t* can be obtained. Among them, the strain *ε_ij_* (t) of SMA wire at any time can be obtained from the lateral displacement difference of the nodes at both ends of the SMA wire, and the strain of the oblique SMA wire arranged in the XY plane of the structure can be obtained by the following formula:

(14) $$\left\{\begin{array}{l} \varepsilon_{ij}(t)=\varepsilon_{kj}(t)=\frac{\sqrt{h^{2}+{\left(w+s_{k}(t)-s_{i}(t)\right)}^{2}}-\sqrt{h^{2}+w^{2}}}{\sqrt{h^{2}+w^{2}}}\;\;\;\;\;s_{k}(t)-s_{i}(t)>0\\ \varepsilon_{ij}(t)=\varepsilon_{kj}(t)=\frac{\sqrt{h^{2}+w^{2}}-\sqrt{h^{2}+{\left(w+s_{k}(t)-s_{i}(t)\right)}^{2}}}{\sqrt{h^{2}+w^{2}}}\;\;\;\;\;s_{k}(t)-s_{i}(t)\leq 0 \end{array}\right.$$

where *h* and *w* are the layer height and single-span span of the structure respectively; when the angle between the *j-*th SMA wire and the positive X direction is less than $90^{{}^{\circ}}$, *S_k_* (t) refers to the lateral horizontal displacement of the *k-*th structural node connected to the upper end of the SMA wire, *S_i_* (t) refers to the lateral horizontal displacement of the *i-*th structural node connected to the lower end of the SMA wire. When the angle between the *j-*th SMA wire and the positive X direction is greater than $90^{{}^{\circ}}$, *S_k_* (t) refers to the lateral horizontal displacement of the *k-*th structural node connected to the lower end of the SMA wire, and *S_i_* (t) refers to the lateral horizontal displacement of the *i-*th structural node connected to the upper end of the SMA wire.

The structural dynamic time history analysis program is compiled to solve the seismic response of the structure. First, the genetic algorithm is employed to optimize the BP neural network constitutive model of SMA wire and the “save” command is used to save the optimized BP neural network model in the form of data structure. Then, the Newmark-β algorithm program is written to solve the dynamic Equation (10), in which the control force *u* can be obtained by calling the BP neural network model optimized by GA.

### 4.2. Optimization Criteria

The sum of the lateral displacements of the structure is taken as the optimized objective function, that is, the optimization criterion is

(15) $$J=\underset{i}{\sum }\left|\Delta_{i}\right|=\underset{i}{\sum }\left|S_{i}-S_{i-1}\right|$$

(16) $$S_{i}=\frac{\sum_{j}S_{ij}}{\sum_{j}j}$$

where *J* is the objective function value; $\left|\Delta_{i}\right|$ represents the absolute lateral displacement of the *i-*th layer; *S_i_* represents the storey drift of the *i-*th layer, and it is the average of the lateral displacement of all nodes of the *i-*th layer; *S_ij_* denotes the absolute lateral displacement of the node *j* of the *i-*th layer. The smaller the objective function value *J*, the smaller the dynamic response of the structure, and the better the control effect of the corresponding SMA wire arrangement on the structure. Based on the basic principles of GA, the following fitness function can be designed:

(17) $$Fit=\frac{1}{J}=\frac{1}{\sum_{i}\left|S_{i}-S_{i-1}\right|}$$

this fitness function meets the design requirements of single value, continuous, non-negative, and maximization. The larger the fitness value corresponding to GA individual, the smaller the objective function value, and the better the corresponding SMA configuration scheme.

### 4.3. Optimization Control and Analysis of Spatial Structure

The spatial model structure is 2 spans along the X direction with each span of 500 mm in length, and along the Z direction is 1 span, the length is 600 mm, the height is 3 layers, and the height of each layer is 500 mm. In order to facilitate the installation and connection of the SMA wire, each layer of 6 nodes has an additional weight of 1 kg, and there are spiral holes for fixing the SMA wire. All rods are Q235 round steel pipes with an outer diameter of 10 mm and a wall thickness of 1 mm. The elastic modulus is 206 GPa, the Poisson’s ratio is 0.3, and the density is 7.85 × 10^3^ kg/m^3^. The following assumptions are made in the analysis of structural seismic response: (a) All masses are concentrated at the nodes of each floor; (b) The initial working temperature of SMA wire is constant in its cross section and length direction.

**Figure 11:**
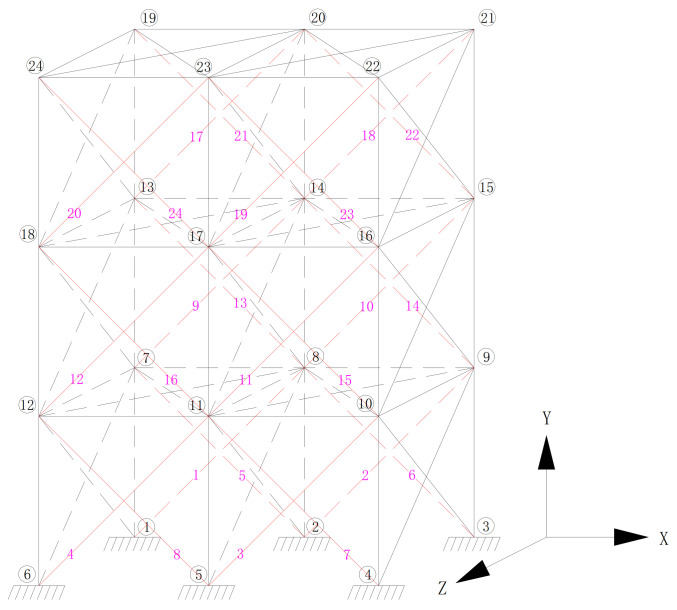
Possible locations of austenitic SMA wires and structural node numbers.

As the vertical and horizontal SMA wires in the XY plane and the SMA wires in the YZ plane have little shock absorption effect on the structure under the action of the horizontal earthquake in the X direction. The SMA wires at these positions are not considered here. Only the diagonal SMA wires in the XY plane are considered, so the node numbers of the spatial model structure and the possible positions of the SMA wires are shown in Figure 11. The positions of the red lines in the figure represent all the possible positions of the SMA wires with a total number of 24.

The operating parameters of GA are set as follows: real-valued coding is used, the initial population size is 40, the maximum number of generations is 50, and the crossover probability is 0.8. When the generation required to maintain the optimal individual unchanged does not exceeds 5, the mutation probability P_m_ is 0.05. When the generation required to maintain the optimal individual unchanged exceeds 20, the mutation probability GP_m_ is 0.2. When the optimal individual does not change and the required generation is between 5 and 20, the mutation probability is taken by linear interpolation between P_m_ and GP_m._

**Figure 12:**
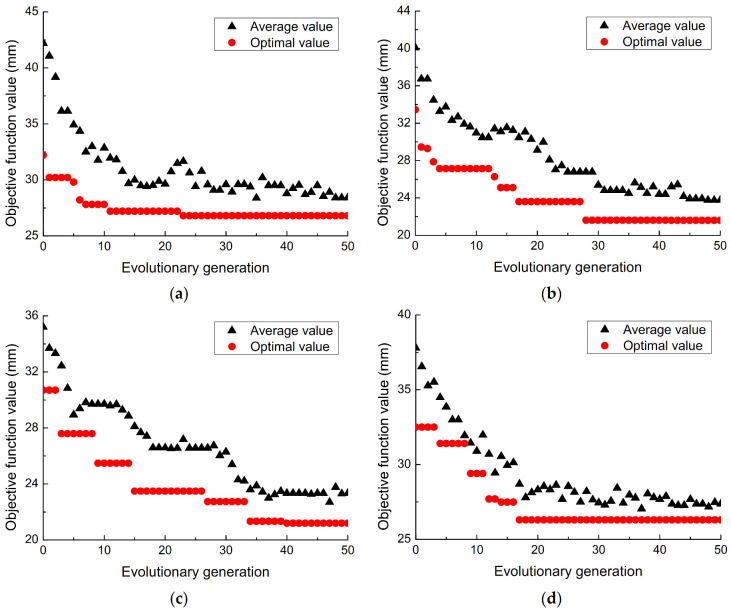
Objective function values with the change of evolutionary generations under different amounts of SMA wire, (**a**) 4 SMA wires, (**b**) 8 SMA wires, (**c**) 12 SMA wires, (**d**) 20 SMA wires.

The El Centro wave with the duration of 20 s, the interval of 0.02 s, and the peak acceleration amplitude of 200 gal is selected as the seismic excitation of the spatial structure along the X direction. In the structure, 2, 4, 6, 8, 12, 16, 20, and 24 SMA wires are arranged for optimizing the position. The change of the objective function value with the evolutionary generation is shown in Figure 12. Table 5 presents the optimal positions of different numbers of SMA wires in the spatial structure, the value of the objective function, and the corresponding shock absorption effects.

**Figure 13:**
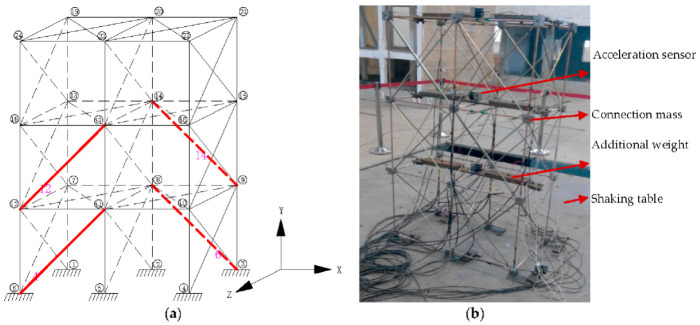
Spatial structure model with 4 austenite SMA wires (**a**) optimal position and (**b**) shaking table test.

It can be seen from the optimization results that with the increase of genetic evolution generations, the optimal value of the objective function gradually decreases, and the average value of the objective function basically shows a gradually decreasing trend, which indicates that the optimal value and the average value are in a gradual convergence. During the process, the value of the objective function changes less and less. The optimized arrangement of the SMA wire has a better suppression effect on the seismic response of the space structure, but it is not that the more the number of SMA wires installed, the better the shock absorption effect. When the number of the SMA wire exceeds a certain number, the shock absorption effect decreases. When 4 austenitic SMA wires are arranged, the SMA wire layout optimized by GA can reduce the storey drift of the structure by 44.51%. Therefore, for the consideration of damping efficiency and economy, 4 SMA wires are selected to passively control the structure. The optimized layout is shown in Figure 13a, and the shaking table test model is shown in Figure 13b.

**Figure 14:**
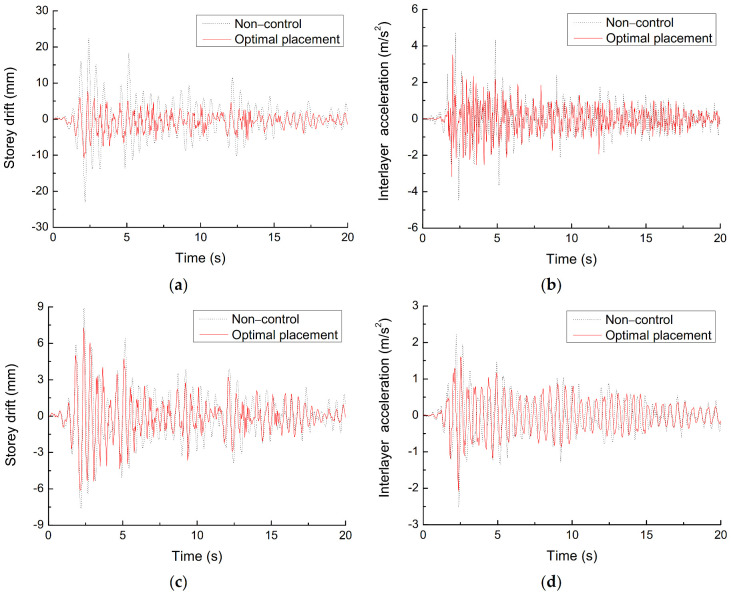
Time history curves of seismic response of spatial model structure with and without control, (**a**) storey drift of first floor, (**b**) interlayer acceleration of first floor, (**c**) storey drift of third floor, (**d**) interlayer acceleration of third floor.

Using node 24, node 18, and node 12 to analyze the acceleration response and displacement response of the structure with and without control, the peak response is shown in Table 6 and Table 7, and the time history curve of storey drift response and interlayer acceleration response is shown in Figure 14. It can be seen that the simulation results are in good agreement with the test results, which supports the rationality and feasibility of MATLAB simulation model for the seismic response analysis of space structure with SMA wires based on BP neural network. Meanwhile, the suppression effect of displacement response is more obvious than that of the acceleration response, the bottom layer has the best damping effect, the storey drift reduction rate is 52.98%, and the interlayer acceleration reduction rate is 25.89%.

## 5. Conclusions

(1) The mechanical tests of SMA wires show that with the increase of the number of cycles, the performance of SMA wires gradually stabilized, the stress–strain curve gradually becomes smooth, the accumulated residual deformation increases gradually, but the residual deformation of single cycle gradually decreases. After 15 cycles, the stress–strain curve tends to be stable, and the residual strain of single cycle is basically 0. With the increase of strain amplitude, the energy dissipation capacity of SMA wires increases obviously. With the increase of loading rate and diameter, the energy dissipation capacity of SMA wires decreases, but not obviously. The strain amplitude is the most prominent factor affecting the energy dissipation capacity of SMA wires.
(2) Taking the material test data of SMA wires as the training sample and test sample of the BP neural network, the BP neural network prediction model optimized by genetic algorithm is established. The simulation results show that the prediction curve of optimized BP neural network is in good agreement with the test curve, and the average absolute percentage error is only 2.13%, the linear coefficient of correlation is 0.9995, and the root mean square error is 5.43. Thus, the model can well reflect the effect of loading velocity on the superelastic properties of SMA wires and is a velocity-dependent dynamic constitutive model with high precision for SMA.
(3) Since the initial weight/threshold is determined by the genetic algorithm, the optimized BP neural network avoids the difference of the prediction model in each run and reduces the phenomenon of network oscillation and non-convergence caused by the improper value of weight/threshold. Compared with the unoptimized BP neural network, it can predict the hysteretic behavior of SMA with better stability and higher accuracy.
(4) The BP neural network optimized by GA was employed to trace the stress–strain curve, and the optimization analysis of the SMA wires in a spatial structure model was carried out under the different seismic excitation. The simulation results are in good agreement with the test results, which supports the rationality and feasibility of MATLAB simulation model for the seismic response analysis of space structure with SMA wires based on BP neural network. Moreover, the results also show that the SMA wires after optimization can effectively reduce the seismic response of the structure, but it is not the case that the more SMA wires, the better the shock absorption effect. When the number of SMA wires exceeds a certain number, the vibration reduction effect gradually decreases. Therefore, the damping effect can be obtained economically and effectively only when the number and location of SMA wires are properly configured. When four SMA wires are arranged, a satisfactory control effect can be gained, the reduction rate of the sum of storey drift can reach 44.51%, and the reduction rate of storey drift and acceleration response at first storey are 52.98% and 25.89% respectively.

## Figures and Tables

**Table 1:** Mechanical properties of SMA wires with different cycles.

Cycles	*σ_a_* (MPa)	*σ_b_* (MPa)	*σ_c_* (MPa)	*σ_d_* (MPa)	Δ*W* (MJ.m^−3^)	*ζ_a_* (%)	*K_s_* (GPa)
1	604.79	604.79	273.75	178.25	6.84	6.11	19.78
2	560.23	572.96	254.65	171.89	6.19	5.81	18.84
3	541.13	560.23	241.92	171.89	5.80	5.44	18.82
5	515.66	541.13	241.92	165.52	5.48	5.18	18.72
10	483.83	509.30	222.82	159.15	5.04	4.76	18.71
15	464.73	496.56	222.82	159.15	4.77	4.48	18.81
20	439.27	483.83	216.45	152.79	4.60	4.37	18.62
25	432.90	477.46	216.45	152.79	4.46	4.18	18.88
30	432.90	477.46	216.45	152.79	4.44	4.16	18.85

**Table 2:** Mechanical properties of SMA wires with different strain amplitudes.

Strain Amplitudes	*σ_a_* (MPa)	*σ_b_* (MPa)	*σ_c_* (MPa)	*σ_d_* (MPa)	Δ*W* (MJ.m^−3^)	*ζ_a_* (%)	*K_s_* (GPa)
3%	432.90	496.56	260.65	120.96	4.46	4.18	18.88
6%	420.17	509.30	254.65	101.86	12.70	6.09	9.21
8%	432.90	515.66	254.65	70.03	20.76	6.60	7.81

**Table 3:** Mechanical properties of SMA wires with different loading rates.

Loading Rates	*σ_a_* (MPa)	*σ_b_* (MPa)	*σ_c_* (MPa)	*σ_d_* (MPa)	Δ*W* (MJ.m^−3^)	*ζ_a_* (%)	*K_s_* (GPa)
10 mm/min	420.17	509.30	254.65	101.86	12.70	6.09	9.21
30 mm/min	426.54	515.36	280.11	107.59	12.31	6.25	8.70
60 mm/min	420.17	502.93	326.04	109.86	11.93	6.15	8.58
90 mm/min	420.17	502.93	331.94	118.23	10.52	5.34	8.71

**Table 4:** Mechanical properties of SMA wires with different diameters.

Diameters	*σ_a_* (MPa)	*σ_b_* (MPa)	*σ_c_* (MPa)	*σ_d_* (MPa)	Δ*W* (MJ.m^−3^)	*ζ_a_* (%)	*K_s_* (GPa)
0.5 mm	483.83	585.69	331.04	203.72	12.43	6.49	8.47
0.8 mm	447.62	527.20	358.10	139.26	12.22	6.01	8.99
1.0 mm	420.17	502.93	331.94	118.23	10.52	5.34	8.71
1.2 mm	349.26	464.20	247.57	70.74	9.63	5.00	8.52

**Table 5:** Optimal results of austenite SMA wires with different amounts.

Number of SMA Wires	Position Optimization Result	Objective Function Value (mm)	Suppression Ratio (%)
0 (non-control)	/	48.3	/
2	4, 15	33.7	30.23
4	4, 6, 12, 14	26.8	44.51
6	2, 6, 7, 13, 14, 20	23.5	51.35
8	4, 7, 8, 13, 14, 15, 21, 24	21.6	55.28
12	2, 8, 9, 10, 11, 13 14, 15, 19, 20, 22, 23	21.2	56.11
16	2, 3, 4, 5, 9, 10, 11, 12, 13 14, 15, 16, 17, 19, 21, 24	22.8	52.80
20	1, 3, 4, 5, 6, 9, 10, 11, 12, 13, 14 15, 16, 17, 19, 20, 21, 22, 23, 24	26.3	45.55
24	All	27.1	43.89

**Table 6:** Peak storey drift and corresponding suppression rates under optimal and uncontrolled conditions.

Floor	Non-Control (mm)		Optimal Placement		Suppression Rate for Simulation Result (%)
	Simulation Result	Test Result	Simulation Result	Test Result	
1	23.052	25.182	10.838	13.431	52.98
2	16.352	15.258	8.702	9.287	46.78
3	8.910	7.641	7.236	6.484	18.79

**Table 7:** Peak interlayer acceleration and corresponding suppression rates under optimal and uncontrolled conditions.

Floor	Non-Control (m/s^2^)		Optimal Placement (m/s^2^)		Suppression Rate for Simulation Result (%)
	Simulation Result	Test Result	Simulation Result	Test Result	
1	4.734	4.407	3.508	3.116	25.89
2	4.028	3.696	3.153	2.519	21.73
3	2.517	1.719	2.068	1.423	17.83

## Data Availability

Not applicable.
